# Research progress in the application of double-lumen endobronchial tubes and bronchial blockers in thoracic surgery

**DOI:** 10.3389/fmed.2026.1849170

**Published:** 2026-08-10

**Authors:** Dan Liu, Qi Zhao

**Affiliations:** Department of Anesthesiology, Sichuan Cancer Hospital & Institute, Sichuan Cancer Center, Affiliated Cancer Hospital of University of Electronic Science and Technology of China, Chengdu, China

**Keywords:** airway management, bronchial blockers, double-lumen endobronchial tubes, one-lung ventilation, thoracic anesthetic management

## Abstract

With the rapid development of thoracic surgery toward minimally invasive and precise approaches, as well as the popularization of video-assisted thoracic surgery (VATS) and robot-assisted video-assisted thoracic surgery (RAVATS), higher requirements have been put forward for one-lung ventilation (OLV) technology during anesthesia. An ideal lung isolation technique not only needs to provide a good surgical field of view but also should minimize the incidence of airway injury, hypoxemia, and postoperative pulmonary complications (PPCs). Currently, double-lumen endobronchial tubes (DLTs) and bronchial blockers (BBs) are the two main tools for achieving OLV. This narrative review summarizes the development status and main surgical types of thoracic surgery in recent years, and analyzes the application of DLTs and BBs in operations such as pneumonectomy, esophageal surgery, and mediastinal surgery. Through a comprehensive comparison of high-quality randomized controlled trials and retrospective studies, the differences between the two airway management devices in terms of lung collapse quality, positioning time, incidence of airway injury, and postoperative recovery indicators are explored. Studies have shown that DLTs have advantages in lung isolation speed and bronchial suction efficiency, while BBs exhibit unique value in difficult airway management, reducing postoperative sore throat, and isolating specific lobes. Moreover, retrospective studies suggest a potential association between BBs use and lower PPCs rates, but this finding requires confirmation by high-quality randomized trials. This article aims to provide evidence-based airway management strategy references for thoracic surgeons and anesthesiologists when facing different surgical types and specific patients.

## 1 Introduction

The core of anesthesia for thoracic surgery lies in the safe and effective implementation of one-lung ventilation (OLV), which ensures adequate oxygenation and ventilation for the patient while providing the surgeon with a motionless and spacious surgical field (1). Since Carlens invented the double-lumen tube in 1949, double-lumen tubes (DLTs) have been the first choice for thoracic anesthesia. However, with the promotion of the enhanced recovery after surgery (ERAS) concept and the advancement of minimally invasive techniques, bronchial blockers (BBs), as a more flexible tool for lung isolation, have been increasingly widely used in clinical practice (2).

In recent years, the controversy over the advantages and disadvantages of DLTs versus BBs has never ceased. On the one hand, DLTs have a large lumen, are relatively easy to operate, and can achieve rapid lung isolation. On the other hand, as an endobronchial device, BBs are used in conjunction with a single-lumen endotracheal tube and offer advantages such as less trauma and the ability to provide mechanical ventilation postoperatively without the need for tube exchange (3). For anesthesiologists, a profound understanding of the performance of these two tools under different surgical approaches is crucial. Starting from the classification of thoracic surgeries, this article systematically reviews the application progress of DLTs and BBs in pulmonary resection, radical esophagectomy, mediastinal tumor resection, and contralateral lung surgery after pneumonectomy, aiming to optimize perioperative airway management decision-making for thoracic surgeons and anesthesiologists.

## 2 Literature search strategy

This study is a narrative review that aims to provide a clinically oriented synthesis of current evidence regarding double-lumen endobronchial tubes (DLTs) and bronchial blockers (BBs) in thoracic surgery. A systematic literature search was conducted to identify relevant studies.

### 2.1 Databases and search period

Literature searches were performed in three electronic databases: PubMed, Web of Science, and Embase, covering publications from January 2010 to June 2025. The reference lists of included articles and relevant review papers were manually screened to identify additional studies that may have been missed by the initial search.

### 2.2 Search terms

The following keywords and their combinations were used: “double-lumen tube,” “double-lumen endobronchial tube,” “bronchial blocker,” “one-lung ventilation,” “single-lung ventilation,” “lung isolation,” “thoracic surgery,” “video-assisted thoracic surgery,” and “anesthesia.” Medical Subject Headings (MeSH) terms were applied when available in PubMed.

### 2.3 Inclusion and exclusion criteria

Inclusion criteria: Peer-reviewed original studies (randomized controlled trials, prospective cohort studies, retrospective cohort studies); Systematic reviews and meta-analyses; Clinical practice guidelines and consensus statements; Studies published in English; Studies reporting at least one of the following outcomes: lung collapse quality, positioning time, airway injury, postoperative pulmonary complications, or postoperative sore throat. Exclusion criteria: Case reports with fewer than 5 patients; Conference abstracts without available full text; Editorials, letters to the editor, and expert opinion articles without original data; Non-English language publications; *In vitro* or animal studies.

### 2.4 Study selection process

Two authors independently screened the titles and abstracts of all retrieved records for eligibility. Full-text articles were then obtained for studies meeting the inclusion criteria and reviewed in detail. Disagreements regarding study eligibility were resolved through discussion and consensus between the two authors.

## 3 Overview of thoracic surgical procedures

Modern thoracic surgery has completely shifted from traditional thoracotomy to minimally invasive surgery represented by endoscopic techniques. The diversification of surgical types directly determines the complexity of anesthetic airway management strategies (4–6).

### 3.1 Pulmonary surgery

In 2022, there were 1.0606 million new cases of lung cancer and 733,300 deaths in China, with the incidence and mortality rates ranking first among all malignant tumors in the country, of which non-small cell lung cancer (NSCLC) accounts for approximately 80 to 85% of all lung cancer cases (7). Currently, pulmonary resection is one of the main treatment methods for NSCLC (8).

Lobectomy plus systematic lymph node dissection is the gold standard for stage I-II NSCLC. However, with the advancement of imaging technology and the popularization of early lung cancer screening, an increasing number of patients are diagnosed with pulmonary lesions through physical examinations at an early stage. Patients with stage Ia disease often undergo segmentectomy or wedge resection with a smaller surgical resection scope, which has extremely high requirements for the quality of lung collapse. Surgeons need to meticulously dissect the intersegmental plane in the collapsed lung tissue (9). The application of video-assisted thoracoscopic surgery (VATS) and robot-assisted thoracoscopic surgery (RATS) prevents surgeons from directly assisting lung collapse by manual compression. Therefore, the “perfect lung collapse” provided by anesthesiologists has become the key to the success of the operation. For patients with tumor lesions involving the bronchus, sleeve lobectomy can be performed to resect the diseased bronchus and lung lobe, followed by bronchial anastomosis to preserve the remaining lung tissue on the same side. This type of surgery requires that the endotracheal tube does not interfere with the surgical field of view. For patients undergoing pneumonectomy, anesthetic management must provide absolute lung isolation to strictly prevent the aspiration of secretions from the affected lung into the healthy lung.

### 3.2 Esophageal surgery

In 2022, there were 224,000 new cases of esophageal cancer in China, accounting for 4.64% of all malignant tumors, and the number of deaths accounted for 7.28% of all malignant tumor deaths, ranking fifth (7). Radical esophagectomy is one of the most complex surgeries in thoracic surgery, usually involving thoracolaparoscopic procedures with operations in the cervical, thoracic, and abdominal surgical fields.

Minimally invasive esophagectomy is often performed in the lateral decubitus position with artificial CO_2_ pneumothorax to assist thoracoscopic procedures, and laparoscopic and cervical operations are performed in the supine position. Due to the close proximity of the esophagus to the posterior wall of the trachea, surgical manipulation can easily interfere with the position of the endotracheal tube and even cause injury to the posterior tracheal wall. Inappropriate selection or malposition of the endotracheal tube may also affect the thoracic surgeon’s exploration of the recurrent laryngeal nerve and dissection of lymph nodes and adipose tissue. Due to the long duration and high trauma of esophageal surgery, some patients require mechanical ventilation support in the intensive care unit (ICU) postoperatively with the tube in place (10).

### 3.3 Mediastinal surgery

Mediastinal tumors (such as thymoma, teratoma, and neurogenic tumors) have a low overall incidence, accounting for less than 1% of thoracic tumors, but their surgical resection carries unique anesthetic risks (7).

Minimally invasive mediastinal tumor resection is often performed in the lateral decubitus, semi-lateral decubitus, or supine position with artificial CO_2_ pneumothorax to assist thoracoscopic procedures. A large anterior mediastinal tumor may compress the main airway or bronchus after anesthetic induction and muscle relaxation, leading to the catastrophic consequence of “neither intubation nor ventilation.” Such surgeries have higher requirements for the speed and safety of airway establishment than the quality of lung isolation itself (11).

### 3.4 Contralateral lung surgery after pneumonectomy

The anesthetic management of this type of thoracic surgery is extremely challenging. It is necessary to fully understand the patient’s general condition, previous surgical type, and the size of the resected lung tissue to assess whether the patient can tolerate conventional OLV. If a patient has previously undergone lobectomy or even pneumonectomy on one side and requires surgical resection for a new or metastatic tumor in the contralateral lung, ventilation solely through the non-operative lung during the operation may fail to maintain normal oxygen saturation. In such cases, selective lobar blockade techniques should be adopted to block only the bronchus of the affected lobe while preserving the ventilation function of other lobes on the same side, or with the support of extracorporeal membrane oxygenation (ECMO) (12).

## 4 Current application status of double-lumen tubes and bronchial blockers

### 4.1 Double-lumen tubes

Since their advent in the mid-20th century, DLTs have evolved from red rubber to polyvinyl chloride (PVC) materials. With the development and widespread clinical application of visualization technology, video double-lumen tubes (Video-DLTs) integrated with a video system have been increasingly used in clinical practice (13).

Although DLTs are available in various models and specifications, their basic structure is consistent: DLTs consist of a main tracheal lumen and a bronchial lumen, each equipped with a tracheal cuff (colorless) and a bronchial cuff (blue). Left-sided DLTs are more widely used clinically due to their relatively easy positioning and high safety. Right-sided DLTs are prone to incomplete lung isolation or endotracheal tube dislocation due to the large variation in the opening position of the right upper lobe bronchus, and are only used under specific indications. DLTs have certain advantages: their large lumen allows for high passive exhaust efficiency and rapid lung collapse; they also facilitate flexible bronchoscopy, negative pressure suction, or continuous positive airway pressure (CPAP) application to both lungs at any time during the operation. Meanwhile, DLTs also have certain limitations: due to their large lumen and relatively hard texture, tracheal intubation is prone to damaging the oropharynx and airway mucosa; intubation is difficult in patients with difficult airways; if the patient needs to be transferred to the ICU for continuous mechanical ventilation postoperatively, it is often necessary to replace the tube with a single-lumen endotracheal tube for convenient airway management, increasing the risk of airway injury.

### 4.2 Bronchial blockers

BBs are slender catheters with an inflatable low-pressure cuff at the tip, inserted through a single-lumen endotracheal tube to block the target bronchus under flexible bronchoscopic guidance.

There are two commonly used types of BBs currently: independent blockers (such as Arndt, Cohen, Fuji) and Y-shaped blockers (such as EZ-Blocker). Independent blockers are inserted inside or alongside the single-lumen tube under bronchoscopic guidance. The Y-shaped blocker has a bifurcated tip that straddles the carina, with two ends entering the left and right main bronchi respectively, achieving lung isolation by inflating one side of the cuff. Its stability is considered superior to that of independent blockers (14). BBs have certain advantages: they are extremely suitable for patients with difficult airways (15), allowing airway establishment with a single-lumen tube first followed by lung isolation; mechanical ventilation can be performed postoperatively without tube exchange for airway management; selective lobar blockade can be achieved; the smaller diameter of the single-lumen tube causes less compression and injury to the airway mucosa. Meanwhile, the use of BBs also has certain limitations: due to the extremely small central lumen of the blocker, lung collapse relies on natural recoil of lung compliance or continuous negative pressure suction, resulting in a slower speed (16); intraoperative position changes or surgical manipulation can easily cause blocker displacement; as the blocker occludes the airway on the surgical side, effective suction of secretions from the affected lung is not possible.

## 5 Application of DLTs and BBs in thoracic surgery

In evaluating the comparative efficacy and safety of DLTs and BBs, it is important to distinguish between evidence from randomized controlled trials (RCTs), which provide the highest level of clinical evidence, and observational studies, which are hypothesis-generating and subject to confounding. The following sections describes device performance within each surgical context; a structured cross-cutting analysis of key clinical outcomes and influencing factors is provided in Section 6.

### 5.1 Application in pulmonary surgery

In conventional lobectomy or segmentectomy, both DLTs and BBs can provide satisfactory OLV to meet surgical needs, but each has its own advantages and disadvantages. A recent meta-analysis including 39 randomized controlled trials (RCTs) showed that the rate of “excellent” lung collapse rating in the DLT group was slightly higher than that in the BB group (RR = 0.94), and the time to achieve complete lung collapse was shorter in the DLT group. This is mainly attributed to the large lumen of DLTs allowing rapid air evacuation (3). Kumar et al. conducted a comparison of left-sided DLTs and BBs in VATS and surprisingly found that the BB group was superior to the DLT group in some lung collapse quality scores, and surgeons could not blindly guess which device was used for anesthesia through the surgical field. This indicates that with skilled operation, BBs can achieve the same clinical effect as DLTs (17). Ruetzler et al. found no statistically significant difference in lung isolation quality between left-sided DLTs and EZ-Blocker, but the incidence of postoperative sore throat and hoarseness was significantly lower in the EZ-Blocker group. There was a statistically significant difference in lung collapse speed between the DLT and BB groups, with the DLT group being 1–3 min faster on average, but this time difference has limited clinical significance in surgeries lasting several hours. The BB group requires longer positioning time due to the need for bronchoscopic guidance to place the blocker in the appropriate position, which may be considered a disadvantage in high-volume surgical centers (18).

In a retrospective propensity score-matched cohort study, Li et al. analyzed thousands of patients undergoing VATS lung cancer surgery and reported that the BB group had lower incidences of postoperative pneumonia (3.9% vs. 11.8%) and overall pulmonary complications (PPCs; 6.6% vs. 16.4%) compared with the DLT group. The authors proposed that reduced airway mucosal trauma from smaller single-lumen tubes and lower driving pressures might underlie this association (19). Similarly, another retrospective cohort study by Liu et al. found lower overall PPCs rates with BBs (25.1% vs. 37.9%) and fewer respiratory tract infections (14.4% vs. 30.3%). There was no statistically significant difference in the incidence of non-pulmonary complications between the two groups (20). Notably, a large propensity score-matched retrospective cohort by Yang et al. found contradictory results: the BBs group demonstrated increased postoperative pulmonary infiltration on the surgical side and a higher ICU admission rate compared with the DLTs group. The authors attributed these findings to several BB-specific issues: (1) unstable positioning leading to intermittent loss of lung isolation and intermittent lung re-expansion during surgery; (2) impaired secretion clearance from the operative lung due to the narrow blocker lumen, potentially promoting atelectasis and infection; (3) longer time to achieve satisfactory lung collapse, which may prolong surgical manipulation time (21). Such discrepancies among retrospective studies highlight that institutional protocols, operator expertise, and patient selection criteria can substantially influence observed outcomes, and they also illustrate why definitive conclusions cannot be drawn from observational data alone.

Almost all meta-analyses consistently show that the incidence of postoperative sore throat, hoarseness, and vocal cord injury is significantly lower in the BB group than in the DLT group (3, 22, 23). For vocalists or patients with high voice requirements, BBs are the first choice for endotracheal intubation in pulmonary surgery.

It should be emphasized that the hypothesis that BBs reduce PPCs has inherent limitations in the strength of evidence, which is primarily supported by retrospective observational data, not by high-quality RCTs. Major confounding factors in these studies include: (1) confounding by indication—patients selected for BBs may differ systematically from those receiving DLTs in terms of airway anatomy, baseline pulmonary function, and comorbidity burden; (2) center and operator experience—centers with higher BB utilization may also have more advanced ERAS protocols or overall better perioperative care; (3) variability in PPC definitions—different studies use varying criteria for pneumonia, atelectasis, and respiratory failure, limiting comparability; (4) publication bias—positive associations may be more likely to be published than negative findings. Large, multicenter RCTs with standardized PPCs definitions are needed to definitively determine whether BBs confer a pulmonary complication benefit over DLTs.

### 5.2 Application in esophageal surgery

With the development of minimally invasive esophagectomy, surgery is often performed in the lateral decubitus position with artificial CO_2_ pneumothorax to assist thoracoscopic procedures, and laparoscopic and cervical operations are performed in the supine position. Due to the high requirements for mediastinal anatomy in esophageal surgery, the selection of an appropriate endotracheal tube facilitates cooperation with the surgeon during the operation. The inflated bronchial cuff of DLTs may deform the left main bronchus or even cause it to protrude posteriorly, affecting the surgeon’s field of view and operating space when dissecting lymph nodes around the left recurrent laryngeal nerve. BBs are inserted through a single-lumen endotracheal tube with minimal distortion of tracheal anatomy, which is more conducive to surgical operations in the posterior mediastinum (24, 25). An RCT of 55 patients undergoing thoracoscopic esophagectomy compared the effects of BBs and DLTs, and the results suggested that BBs were superior to DLTs in lung collapse scores at 5 and 10 min after pleural incision, which may be related to the assistance of artificial CO_2_ pneumothorax (26). In addition, if the tracheal membrane is torn due to traction during esophageal surgery, the large lumen of DLTs may aggravate the tear or hinder repair; while using BBs in conjunction with a single-lumen endotracheal tube, the blocker can be quickly withdrawn in the event of airway injury, providing space for surgical repair. In a retrospective propensity score-matched cohort study, Li et al. compared the application of OLV and two-lung ventilation in thoracoscopic esophagectomy. Although the main comparison was of ventilation modes, it also indirectly pointed out that hypoxemia and high airway pressure caused by traditional DLTs are reasons for surgeons to switch to intraoperative two-lung ventilation or BB replacement (27).

### 5.3 Application in mediastinal surgery

For large anterior mediastinal tumors, the core of anesthesia is to prevent airway collapse. For patients at risk of airway compression, awake intubation with preserved spontaneous breathing is the first choice (6). A single-lumen endotracheal tube is easier to pass through the airway stenosis than a DLT. Once the single-lumen tube is successfully inserted and passes the stenosis, inserting a BB for lung isolation is a safer strategy than directly attempting DLT insertion (11, 28). For conventional thoracoscopic mediastinal tumor resection without airway compression, a network meta-analysis showed that video-DLTs were superior to BBs in positioning speed, but the incidence of postoperative sore throat was significantly lower in the BB group than in the DLT group (29).

### 5.4 Application in contralateral lung surgery after pneumonectomy

In 2024, Jeong et al. reviewed 13 cases of contralateral lung resection after pneumonectomy, with no perioperative deaths but a complication rate as high as 36%. The use of BBs for selective lobar blockade is the preferred airway management strategy for such surgeries (12). For example, a patient who has undergone left pneumonectomy requires right upper lobectomy. The anesthesiologist can accurately position the BB to the opening of the right upper lobe bronchus. During the operation, the right upper lobe is blocked, and mechanical ventilation is continued to the right middle and lower lobes through the single-lumen endotracheal tube. This not only ensures the surgical field but also preserves approximately 2/3 of the ventilation area to maintain oxygenation. For patients who cannot tolerate any lobar blockade, ECMO provides safety and allows complete apnea during the operation (30).

### 5.5 Application in lung transplant surgery

Lung transplantation represents a unique and challenging scenario for airway management, with distinct requirements during the implantation, and reperfusion phases. Both DLTs and BBs have roles in this setting, though DLTs remain the conventional standard. DLTs have long been the gold standard for lung transplantation, DLTs can provide continuous ventilation to the native lung during implantation of the donor lung, thereby maintaining stable intraoperative oxygenation. Furthermore, they do not require cuff placement in the surgical-side bronchus, therefore, no interfere with procedures such as bronchial anastomosis. After bronchial anastomosis and reperfusion of the donor lung, the DLT allows selective ventilation of the newly implanted lung to assess compliance, gas exchange, and anastomotic integrity before completing chest closure. The large bronchial lumen enables effective clearance of blood and secretions from the operative field, which is particularly important during implantation and reperfusion when bleeding and edema fluid can accumulate.

In bilateral sequential lung transplantation, DLTs are nearly universally preferred. The ability to alternate ventilation between lungs as each side is transplanted is essential. BBs are generally not suitable for bilateral transplant because the blocker would need to be repeatedly repositioned from side to side, disrupting surgical workflow and increasing the risk of hypoxemia during transitions.

### 5.6 Application in thoracic surgery with VV-ECMO support

Veno-venous extracorporeal membrane oxygenation (VV-ECMO) provides systemic oxygenation and carbon dioxide removal independent of native lung function, fundamentally transforming the goals of intraoperative airway management. When VV-ECMO is employed, the primary purpose of the airway device shifts from maintaining gas exchange to optimizing the surgical field and protecting the airways.

BBs are often the preferred airway device in VV-ECMO-assisted thoracic surgery. Since oxygenation is provided by ECMO, the goal of lung isolation is purely to create a quiet, collapsed surgical field. BBs achieve effective lung isolation without the bulk of a DLT, providing more working space in the trachea and carina region. For tracheal resection and anastomosis, a single-lumen tube with BB can be advanced or withdrawn across the surgical field as needed. After complex surgery, patients typically remain intubated and on VV-ECMO in the ICU. The single-lumen tube is already in place for continued ventilatory support, avoiding the need for tube exchange.

An important nuance of VV-ECMO anesthesia is that complete apnea can lead to progressive lung atelectasis and increased pulmonary vascular resistance, which may affect right ventricular function. DLTs allow selective recruitment of each lung, whereas with BBs, the blocked lung remains collapsed until the blocker is deflated. This difference may influence device selection depending on the anticipated duration of apnea and the patient’s baseline pulmonary hemodynamics.

## 6 Comparative analysis of key clinical outcomes

This section synthesizes the available evidence on three core outcome domains: oxygenation and hypoxemia, lung collapse quality in different surgical settings, and device failure rates. The influence of operator experience and institutional practice patterns is also discussed.

### 6.1 Oxygenation and hypoxemia rates

Intraoperative hypoxemia remains one of the most feared complications of one-lung ventilation (OLV). Available evidence suggests that the risk of hypoxemia is broadly similar between DLTs and BBs when devices are correctly positioned, but the mechanisms and management of desaturation events are different.

Most RCTs have found no significant difference in intraoperative SpO_2_ nadir or the incidence of desaturation events (SpO_2_ < 90%) between the two devices in patients with normal pulmonary function undergoing elective lobectomy (31–33). A 2023 meta-analysis by Kumar et al. reported no statistically significant difference in the rate of intraoperative hypoxemia between left-sided DLTs and EZ-Blockers (RR 1.12, 95% CI 0.78–1.61) (17).

A key advantage of DLTs in managing hypoxemia is the ability to rapidly apply continuous positive airway pressure (CPAP) to the non-ventilated lung or perform intermittent recruitment maneuvers through the dedicated bronchial lumen. With BBs, applying CPAP to the operative lung is more difficult because the blocker’s central lumen is very narrow, limiting gas flow; in practice, anesthesiologists must partially withdraw the blocker to apply CPAP, which may lose lung isolation. For patients with marginal pulmonary function or those requiring selective lobar blockade, BBs offer a unique oxygenation advantage by preserving ventilation to non-operative lobes—a strategy that is not possible with standard DLTs, which isolate the entire lung. In this specific population, BBs are associated with substantially lower hypoxemia risk and may avoid the need for extracorporeal support.

### 6.2 Lung collapse quality across surgical contexts

Lung collapse quality is not a uniform endpoint—it depends heavily on the surgical approach, the use of CO_2_ pneumothorax, and the precision required for the procedure.

In conventional VATS lobectomy without CO_2_ insufflation, most RCT studies agree that DLTs achieve faster and more complete initial lung collapse due to the large-bore bronchial lumen allowing passive egress of air, DLTs achieve satisfactory collapse 1–3 min faster than BBs. However, this time difference is of limited clinical significance in procedures lasting 2–4 h. The final quality of collapse after 10–15 min is generally comparable between the two devices (31–33).

In surgeries using CO_2_ pneumothorax (e.g., minimally invasive esophagectomy, subxiphoid mediastinal surgery, some robotic procedures). Artificial CO_2_ insufflation actively compresses the lung, equalizing the collapse advantage of DLTs. A RCT study performed by Zhang et al. have even found superior collapse scores with BBs in CO_2_-aided thoracoscopy, possibly because the single-lumen tube causes less anatomical distortion of the bronchial tree, allowing more uniform lung deflation under positive pressure (26).

### 6.3 Device failure rates

Device failure is a clinically important but underreported endpoint, which is defined as malposition requiring repositioning, loss of isolation during surgery, or emergency conversion to an alternative device. DLTs have a relatively shallow learning curve. Most anesthesiologist can achieve competent DLTs placement after 10–20 supervised procedures, and success rates plateau quickly. DLTs malposition typically occurs at the time of intubation or with patient repositioning, once correctly positioned, DLTs are relatively stable due to their size and shape; intraoperative displacement requiring mid-procedure repositioning is uncommon.

BBs have a steeper learning curve that is closely tied to bronchoscopy proficiency. The learning effect is substantial; novice operators may take 2–3 times longer to position BBs and experience higher displacement rates compared with experts. BBs displacement is more frequently reported, particularly with independent blockers (21). BBs displacement is often transient and can be corrected with bronchoscopy repositioning, but each adjustment interrupts the surgical workflow and carries a small risk of hypoxemia during the repositioning period. Emergency conversion rates from one device to the other are very low in experienced anesthesiologist.

Institutional culture and equipment availability also heavily influence device selection. Many centers have a historical preference for DLTs and have standardized protocols around them, making BBs adoption slow even when evidence suggests equivalence or advantages in specific populations. Conversely, centers with robust ERAS programs and extensive experience with difficult airway management often prefer BBs as their default approach, and their outcomes reflect this accumulated expertise.

## 7 Economic and cost-effectiveness analysis

In the medical decision-making process, the primary goal is to achieve the desired clinical effect. In addition, medical cost is also a factor that anesthesiologists need to consider when selecting airway devices. A cost-effectiveness analysis study showed that although the unit price of video-DLTs is higher, due to the reduced intraoperative demand for reusable flexible bronchoscopes and lower costs for cleaning, disinfection, maintenance, and procurement, they are more cost-effective than traditional DLTs plus reusable bronchoscopes in terms of comprehensive cost, saving approximately $51 per operation. BBs themselves are relatively expensive and must be used with disposable or reusable bronchoscopes, so their direct consumable cost is usually higher than that of traditional DLTs. However, in patients requiring postoperative intubation, mechanical ventilation can be achieved by removing the blocker without the need for reintubation, avoiding the cost and risk of secondary intubation, thus having potential health economic advantages (34).

## 8 Discussion in modern thoracic anesthesia

Airway device selection is not an isolated decision; it must be integrated within the broader framework of contemporary thoracic anesthesia and perioperative care.

Non-intubated video-assisted thoracic surgery (NIVATS) avoids endotracheal intubation entirely, maintaining spontaneous ventilation under regional anesthesia combined with conscious sedation. The primary advantages include avoidance of OLV-related physiological stress, reduced ventilator-induced lung injury (VILI) risk, faster postoperative recovery, and shorter hospital stay. Furthermore, intraoperative conversion to conventional OLV is required in approximately 5–15% of cases due to hypoxemia, surgical complexity, or patient discomfort (35). In this scenario, BBs may offer an advantage, if the patient already has a laryngeal mask airway, BBs can be rapidly inserted to achieve lung isolation. This strategy is increasingly recognized as a valuable backup protocol in NIVATS.

The choice between total intravenous anesthesia (TIVA) and inhalational anesthesia has important interactions with one-lung ventilation physiology. Volatile anesthetics are known to dose-dependently attenuate hypoxic pulmonary vasoconstriction (HPV), which could theoretically worsen oxygenation during OLV. Propofol-based TIVA, by contrast, is generally considered to have minimal effect on HPV. The choice between DLTs and BBs appears to have little association with the effects of anesthesia on HPV. However, one detail deserves attention: during the early phase of one-lung ventilation, the use of BBs may result in a slightly slower collapse of the operative lung and may also increase the risk of atelectasis. Theoretically, this could amplify differences related to HPV among different anesthetic techniques; however, no specific study has confirmed this.

Lung-protective ventilation (LPV) has become the standard of care during OLV, supported by evidence that it reduces postoperative pulmonary complications and improves outcomes (36). Core LPV principles include low tidal volume, positive end-expiratory pressure titration, recruitment maneuvers, permissive hypercapnia and driving pressure limitation. BBs are used through standard single-lumen endotracheal tubes, which typically have lower overall resistance than the bronchial lumen of DLTs. This means the peak and driving pressures may be slightly lower with BBs in equivalent tidal volumes, potentially facilitating LPV implementation. However, this theoretical advantage is small in adults with normal lung function. DLTs uniquely allow independent ventilation strategies for each lung, which could apply CPAP to the operative lung while maintaining LPV. BBs cannot provide true differential ventilation because the blocker simply occludes the bronchus.

Understanding ventilator-induced lung injury (VILI) mechanisms helps for airway device choice, which might influence postoperative pulmonary outcomes. The classic paradigms of VILI include volutrauma, atelectrauma, barotrauma and biotrauma (37). As noted above, BBs may produce slightly lower driving pressures for equivalent ventilation, which may theoretically reduce VILI risk. DLTs with their larger diameter and bronchial cuff, cause greater direct mechanical trauma to the tracheobronchial mucosa. This local injury may trigger a mild inflammatory cascade that may theoretically amplify biotrauma from ventilation. BBs produce slower lung collapse and may result in more gradual atelectasis of the operative lung compared with the rapid deflation achieved with DLTs.

Intraoperative hemorrhage is an infrequent but critical complication in thoracic surgery. The choice of airway device significantly impacts the anesthesiologist’s ability to manage bleeding and protect the non-operative lung from aspiration of blood. DLTs have a dedicated, large-bore bronchial lumen (typically 6–7 mm internal diameter for adult sizes) that accommodates standard suction catheters. Blood and clot can be rapidly evacuated from the operative bronchus and lung, maintaining visualization and preventing accumulation. This is particularly valuable in procedures at higher bleeding risk. BBs have an extremely narrow central lumen (typically 1–2 mm), which is inadequate for suctioning blood—especially clotted blood. The blocker physically obstructs the bronchus, preventing any meaningful suction access to the operative lung through the airway. If significant bleeding occurs, the blocker must be fully withdrawn to allow suctioning, and the single-lumen tube must be advanced into the main bronchus of the non-operative side to protect it from aspiration. This emergency conversion takes time and carries a risk of hypoxia and aspiration during the maneuver. For surgeries with anticipated high bleeding risk, DLTs are generally preferred due to their superior suction capability and more reliable lung isolation security. BBs may still be appropriate for lower-risk procedures in patients where other factors (e.g., difficult airway) take priority, but the team should be prepared for emergency airway conversion if significant bleeding occurs.

Enhanced Recovery After Surgery (ERAS) protocols for thoracic surgery emphasize a multimodal approach to minimize physiological stress and accelerate recovery. Airway device selection is one component of this broader strategy. Most randomized trials in elective lobectomy populations find no significant difference in time to extubation between DLTs and BBs when immediate extubation is the goal (38, 39). Both devices are compatible with ERAS protocols, and patients are typically extubated in the operating room or within 1–2 h postoperatively regardless of device type. BBs may lead to lower incidence of postoperative sore throat and hoarseness, avoiding the physiological stress of tube exchange for postoperative ventilation. DLTs more reliable lung isolation may reduce operative time in high-throughput settings, the better secretion clearance may reduce postoperative atelectasis and pneumonia risk in patients with heavy sputum burden. Optimal ERAS outcomes depend on integrating device selection with other evidence-based strategies: lung-protective ventilation, goal-directed fluid therapy, regional analgesia, early mobilization, and standardized postoperative care pathways. The choice of airway device should be tailored to the individual patient and procedure within this multimodal framework.

## 9 Conclusion and clinical recommendations

The choice between DLTs and BBs should not be viewed in isolation but as one component of a comprehensive perioperative strategy that includes lung-protective ventilation, appropriate anesthetic technique, regional analgesia, and ERAS principles. Modern developments such as non-intubated surgery further expand the airway management toolkit for selected patients. DLTs have many advantages: they can provide definite and reliable lung isolation, physically separate the left and right lungs, and are the absolute indication for lung abscess and pulmonary hemorrhage to prevent the flow of pus and blood from the affected lung to the healthy lung. DLT positioning is relatively simple and rapid, and skilled anesthesiologists can perform blind intubation successfully. Moreover, Video-DLTs can realize the “plug-and-view” function, greatly shortening the positioning time. DLTs have a relatively large lumen, allowing the use of large suction catheters for negative pressure suction to effectively remove thick secretions. DLTs allow flexible selection of ventilation modes and facilitate the implementation of CPAP for the single lung or intermittent ventilation of both lungs. Meanwhile, DLTs also have certain disadvantages: due to their large lumen, the risk of airway injury is high, and the probability of tracheal tear and vocal cord injury is significantly higher than that of BBs. They are less suitable for difficult airways, as the large and pre-shaped tube is difficult to pass through patients with glottic stenosis or limited mouth opening. If the patient needs to be transferred to the ICU for mechanical ventilation support, it is usually necessary to replace the tube with a single-lumen endotracheal tube, which carries the risk of hypoxemia and airway loss.

BBs are the first choice for airway management in patients with difficult airways. An artificial airway can be established first through a small-diameter single-lumen endotracheal tube, followed by insertion of a BB to achieve OLV, with the entire process being safe and controllable. Patients using BBs have a lower incidence of postoperative sore throat and hoarseness, which is in line with the contemporary ERAS concept. BBs can achieve selective blockade and are the only device capable of lobar-level isolation, making them the first choice for patients undergoing re-pulmonary resection after pneumonectomy and some patients with extremely poor pulmonary function. After the operation, only the blocker needs to be removed, and the single-lumen tube can be retained for transfer to the ICU for mechanical ventilation, avoiding the risk of tube exchange. Meanwhile, BBs also have certain disadvantages: intraoperative position changes or surgical traction of the hilum can easily cause blocker displacement, leading to isolation failure and requiring repeated adjustments. BB positioning is time-consuming and must rely on bronchoscopic guidance, with high requirements for bronchoscopic operation skills. Generally, after using a blocker, air evacuation on the surgical side is slow, and lung collapse takes a long time unless assisted by artificial CO_2_ pneumothorax or combined with negative pressure suction techniques.

**Figure 1:**
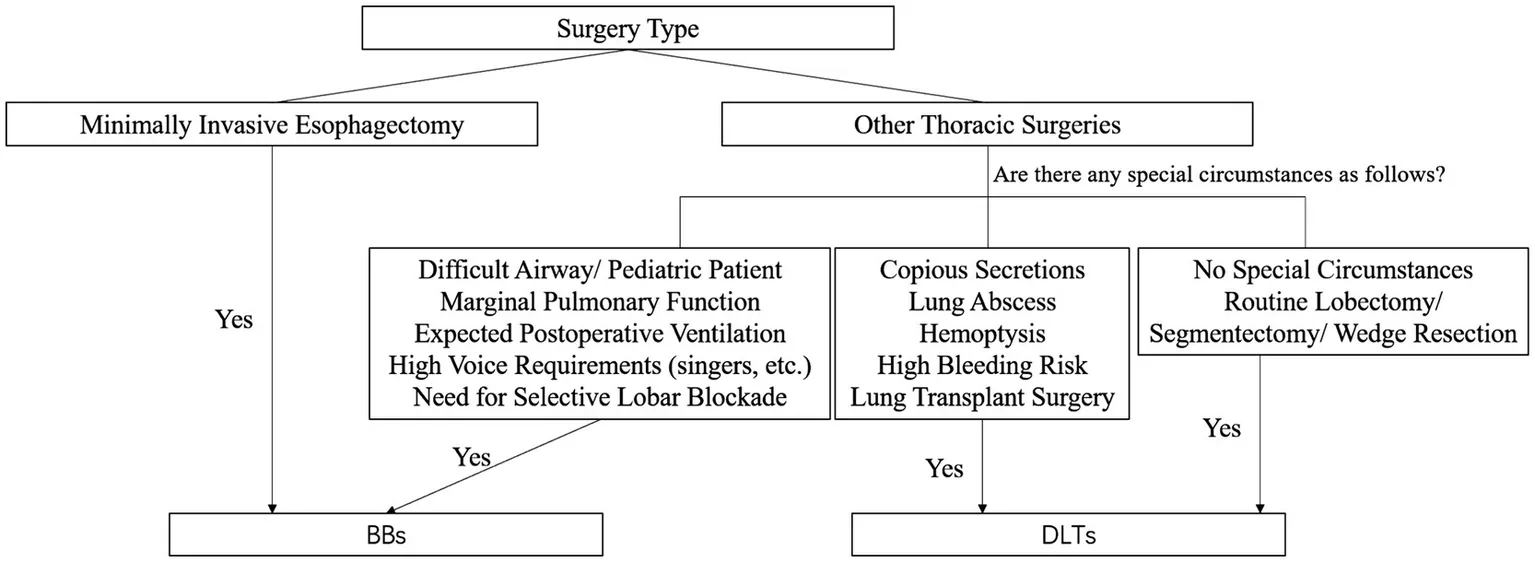
Decision-making flowchart for airway device selection.

Based on the existing literature and research progress, DLTs and BBs each have their own merits, with no absolute superiority or inferiority, but rather complement each other. Table 1 provides a comparative summary of their comprehensive performance. Figure 1 presents a decision-making flowchart for airway device selection, considering various surgical procedures and patients’ specific factors. Thoracic anesthesiologists should select appropriate tools for different patients during anesthetic management to ensure perioperative safety.

1. Conventional lobectomy and segmentectomy: DLTs are recommended as the first choice due to their rapid lung collapse, convenient suction, and reliable fixation, which best meet the needs of high-volume surgeries. Video-DLTs are recommended for eligible patients.
2. Thoracic surgery in patients with difficult airways: The use of a single-lumen endotracheal tube combined with BBs is strongly recommended to safely establish the airway first, followed by OLV according to surgical needs.
3. Esophageal surgery: BBs are preferred due to their minimal impact on tracheal anatomy and convenience for postoperative mechanical ventilation management.
4. Patients with marginal pulmonary function or after pneumonectomy: BBs must be used for selective lobar blockade to maximize the preservation of ventilation area and maintain oxygenation.
5. Pediatric thoracic surgery: Since the smallest model of DLTs (26Fr) is only suitable for children over 8–10 years old, BBs (5Fr, 7Fr) are the only choice for achieving OLV in younger children (40).

**Table 1:** Comprehensive performance comparison of DLTs and BBs.

Comparison aspect	DLTs	BBs
Lung collapse quality	Excellent (faster collapse, more complete suction)	Good (slower, requires suction assistance)
Positioning time	Fast (high success rate of blind intubation; faster with Video-DLTs)	Slow (must be guided by fiberoptic bronchoscopy)
Incidence of postoperative sore throat	High (larger diameter, greater rigidity)	Low (smaller diameter, less irritation)
Incidence of postoperative pulmonary complications	Relatively high (may be related to airway inflammation)	Relatively low (reduces airway trauma)
Suitability for difficult airway	Poor (difficult to pass through a narrow glottis)	Excellent (can be used with a single-lumen tube)
Selective lobar isolation	Not available (only whole lung isolation)	Available (e.g., right upper lobe, right lower lobe)
Postoperative mechanical ventilation	Requires tube exchange (high risk)	No tube exchange needed (simply withdraw the blocker)
Equipment cost	Low/Medium (higher for Video-DLTs)	High (requires fiberoptic bronchoscopy and single-lumen tube)

In summary, thoracic anesthesiologists should be proficient in both techniques. Future research will focus on the design of novel anti-displacement BBs, the development of visualized BBs, and artificial intelligence-assisted airway management decision-making systems to further improve the safety and precision of thoracic anesthesia and safeguard patients’ perioperative safety.
